# A centriole's subdistal appendages: contributions to cell division, ciliogenesis and differentiation

**DOI:** 10.1098/rsob.200399

**Published:** 2021-02-10

**Authors:** Nicole A. Hall, Heidi Hehnly

**Affiliations:** Department of Biology, Syracuse University, Syracuse NY, USA

**Keywords:** centrosome, subdistal appendages, division, cilia, ciliopathies, midbody

## Abstract

The centrosome is a highly conserved structure composed of two centrioles surrounded by pericentriolar material. The mother, and inherently older, centriole has distal and subdistal appendages, whereas the daughter centriole is devoid of these appendage structures. Both appendages have been primarily linked to functions in cilia formation. However, subdistal appendages present with a variety of potential functions that include spindle placement, chromosome alignment, the final stage of cell division (abscission) and potentially cell differentiation. Subdistal appendages are particularly interesting in that they do not always display a conserved ninefold symmetry in appendage organization on the mother centriole across eukaryotic species, unlike distal appendages. In this review, we aim to differentiate both the morphology and role of the distal and subdistal appendages, with a particular focus on subdistal appendages.

## Graphical abstract

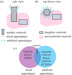

## 1. Introduction: what is conserved across eukaryotes in centrosome structure?

**Figure 1.**
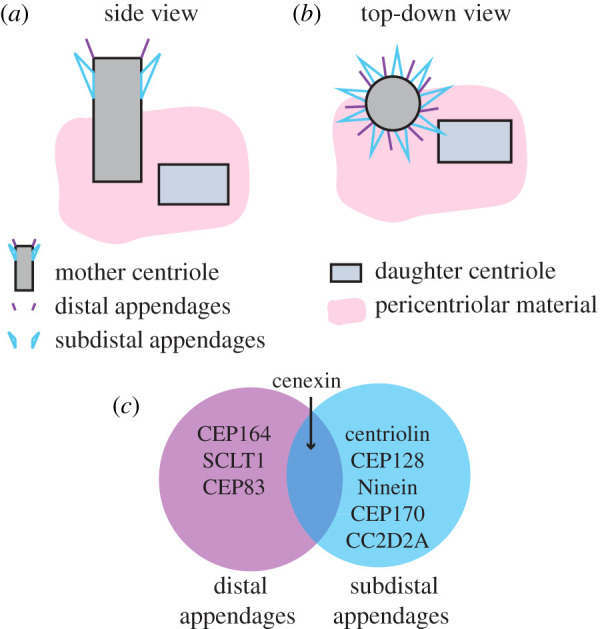
The centrosome comprises two centrioles which inherently differ in both age and structure. (*a*) Side view of a human retinal pigment epithelial cell centrosome schematic with the oldest centriole (mother, grey barrel with blue and purple appendages) and the younger centriole (daughter, light blue barrel devoid of appendages) surrounded by pericentriolar material (PCM, pink). (*b*) Top-down view of centrosome schematic with the oldest centriole presenting with ninefold symmetry of both distal (purple) and subdistal* (blue) appendages. *Note: We are depicting ninefold symmetry of the subdistal appendages found within human retinal pigment epithelial cells, but subdistal appendage number can change due to extracellular cues and vary across cell types within a single species. (*c*) Venn diagram highlighting a number of proteins known to localize to either distal appendages (purple), subdistal appendages (blue) or both appendage structures (denoted with arrow pointing to overlapping region).

The centrosome is a structure that modulates key cellular processes ranging from proper mitotic spindle placement to the construction of a functional cilium. The centrosome comprises two barrel-like structures, called centrioles. Centrioles are approximately 450–550 nm in length and 250 nm in outer diameter that contain cylindrical arrays of triplet microtubules organized with ninefold radial symmetry. Centrioles are polarized along their proximal-to-distal axis. The proximal region is defined by the presence of a cartwheel structure, which serves as the seed for centriole formation and imparts the ninefold symmetry to the centriole (reviewed in [1–3]). What is striking about the two centriole barrels is that one is structurally distinct at the distal end, compared to the other, where it can sometimes contain two sets of appendage-like structures called subdistal and distal appendages (figure 1*a*,*b*). When distal appendages are present, they have a ninefold symmetry, whereas subdistal appendage organization is varied across cell types and species. In animals, the centrioles are encompassed by a matrix consisting of large coiled-coil proteins of the pericentrin family, which anchor other matrix proteins [1–3]. The combination of all these matrix proteins is referred to as the pericentriolar material (PCM). Excluding centriole appendages, the centriole molecular composition and structure is predominantly conserved across the eukaryotic tree of life. While numerous studies have discussed the centriole, PCM and distal appendage ultrastructure, what has not been thoroughly discussed is the subdistal appendage molecular components and their structural organization in different cellular contexts across organisms throughout development.

*Caenorhabditis elegans* and *Drosophila melanogaster* both lack distal and subdistal appendages, whereas the single-cell eukaryote *Chlamydomonas reinhardtii* contains transitional fibres organized into a ninefold symmetry that probably play a similar role as distal and subdistal appendages of vertebrate cells [4]. Vertebrate cells consistently display both distal and subdistal appendages, identified in species ranging from zebrafish [5] to mammalian cells [6] (figure 1). The distal appendages, composed of a handful of proteins including CEP164, SCLT1 and CEP83 (figure 1*c*), act as a barrier between the mother centriole and the ciliary axoneme. This barrier has been shown to be crucial in both the docking of the centriole to the membrane and regulating ciliary axoneme growth [7]. In addition, centrioles exhibit a ninefold symmetrical ultrastructure which translates to a ninefold symmetry of the distal appendages. What is fascinating is that unlike the highly conserved symmetry of the distal appendages, the conservation of subdistal appendage symmetry is not so clear. For example, across different human cell lines there is a disparity in appendage number. Human retinal pigment epithelial cells present with a ninefold symmetry in subdistal appendages [8] (figure 1*b*), but aorta endothelial cells have one to four subdistal appendages and umbilical vein endothelial cells have three to six subdistal appendages. Human aorta endothelial cells, unlike human umbilical vein endothelial cells, can adjust their subdistal appendage number due to extracellular cues [9]. These findings suggest several possibilities, one of which is that subdistal appendage symmetry can be impacted by extracellular signals that have the potential to effect centrosome function. An additional outcome that these studies present is that subdistal appendage number within a single mother centriole can vary across cell types within a single species. One question remains: why are the subdistal appendages so variable in number across species and cell types? By understanding the changes centrosome structure undergoes during the cell cycle, the molecular composition of appendages, and the interactions that subdistal appendage proteins may have during different cell cycle stages and in different cell types, we may start to answer this question.

## 2. The cell cycle and centriole appendages

**Figure 2.**
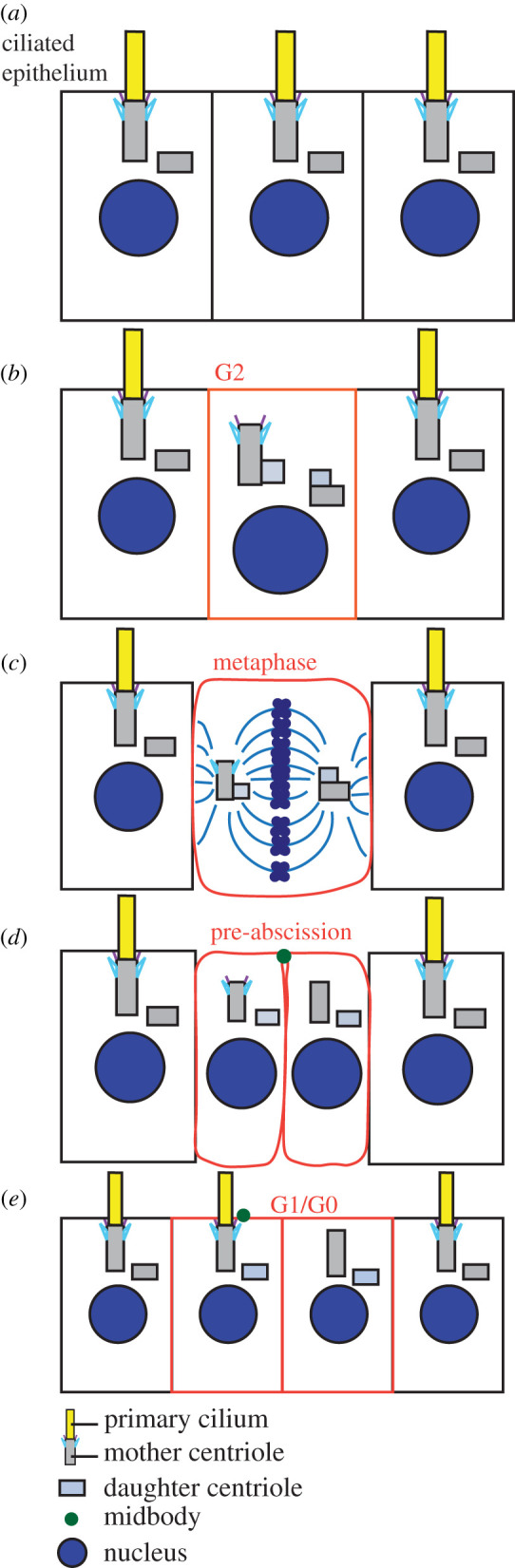
The cytokinetic midbody directing ciliogenesis. (*a*) Ciliated epithelium with cilia at the apical membrane. (*b*) When cells decide to enter division and duplicate their centrosomes the cilia are disassembled, and the centrioles duplicate to make two mitotic spindle poles. With one mitotic centrosome containing the oldest centriole (grey barrel with blue and purple appendages, noted as the mother) that has associated appendages. (*c*) The two mitotic centrosomes that are inherently asymmetric due to centriole age assemble the bipolar microtubule-based spindle. (*d*) As cells progress through anaphase and cytokinesis a cytokinetic bridge is formed with an associated midbody (green dot). (*e*) As the cells abscise the bridge the midbody can still be attached and marks a place on the apical membrane where the cell with the oldest mother centriole will grow a cilium first.

The centrosome constrains a pair of centrioles where one centriole is inherently older (mother) than the other (daughter). The two centrioles are surrounded by a matrix of PCM. The PCM contains hundreds of proteins, that include cell cycle regulators, signalling molecules and microtubule organizers making it the dominant microtubule organizing centre in many cell types. These components of the centrosome allow it to play a fundamental role in the establishment of the mitotic spindle, necessary for chromosomal segregation and cilia formation (figure 2). To establish a bipolar mitotic spindle, a single centrosome needs to be duplicated into two mitotic centrosomes during S phase where the mother and daughter centrioles template a procentriole that forms orthogonally from the parent centriole in a Sas-4 dependent fashion ([10], figure 2*b*). The resolution of the orthogonally orientated centrioles occurs by the end of mitosis where the two centrioles can organize into independent centrosomes. However, of the four centrioles between the two centrosomes one is inherently the oldest and maintains certain components that are essential for establishment of mother centriole appendages (figure 2*b*,*c*). An example of this is the subdistal and distal appendage protein, cenexin (figure 1*c*). Cenexin has clearly been identified on the oldest centriole from G2 through mitosis [11–13], whereas other subdistal appendage proteins—ninein and centriolin—tend to reorganize to the PCM as a cell enters mitosis [14]. A distal appendage protein, Cep164 (figure 1*c*), while present at G1, seems to be lost as cells enter mitosis [15]. These studies suggest that while aspects of subdistal appendages may remain at the oldest centriole as cells proceed through division, distal appendages and components of the subdistal appendages are resourced to other locales. Once division is complete, each daughter cell's centrosome containing either the original daughter centriole or original mother centriole will undergo a series of biochemical and structural developments required for appendage formation and pericentriolar reorganization that is needed for cilia development. However, this process does not seem to occur in the same time frame for the two independent centrosomes that form within the two daughter cells, suggesting a distinction in cilia formation timing based on centrosome age [16] (figure 2*e*).

A study in cultured cells highlighting the possible disparity in appendage formation emphasized that daughter cells formed post division make cilia at different rates. Following division, the daughter cell that receives the oldest mother centriole grows the primary cilium first (figure 2*d*,*e*) and in some cases can grow a primary cilium while staying interconnected by a cytokinetic bridge [16]. Asynchronous cilium formation was also observed following neuronal stem cell divisions in the mouse neural tube [17]. Once the two daughter cells completely form primary cilia there is an asymmetric recruitment of signalling proteins, inversin and PDGFRa, to the primary cilium at the oldest mother centriole [16]. These findings have important implications for appendages where they are probably contributing to cargo sorting events that define how a cilium is built and what is going to reside within it. Since these studies, we have gained important information that an endocytic compartment, the recycling endosome, can directly interact with subdistal appendages of the mother centriole [18]. Recycling endosomes, or the endocytic recycling compartment, is an endocytic compartment organized in a peri-centrosome region that returns cargo proteins back to the plasma membrane [19]. The recycling endosome is also implicated in regulating ciliogenesis [20,21] and polarity formation [22]. Recycling endosome organization and ability to sort cargo is modulated by the small GTPase Rab11, which has been identified in a network of regulators required for cilia formation through its interaction with the guanine exchange factor (GEF), Rabin8 [20,21]. Rab11 is thought to first be recruited to a pericentriolar region where it brings with it Rabin8 to activate another small GTPase, Rab8. At this point, Rab8 can work to construct a nascent cilium [20,21]. Thus, it is exciting to postulate that mature subdistal appendages are required to recruit Rab11-positive endosomes to the mother centriole to initiate a GTPase cascade between Rab11 and Rab8 commencing ciliogenesis.

The temporal relationship of appendage formation in relation to the cell cycle has not been identified and will probably provide mechanistic insight to how appendages are built, their function as a structure, and needed information about the individual proteins required for its assembly. Studies in human cervical cancer cells (HeLa) demonstrated a lack of both subdistal and distal appendages in metaphase cells using serial transmission electron microscopy approaches [23]. However, when following molecular components that make up either subdistal, distal, or both appendages things become interesting. For instance, the subdistal and distal appendage protein cenexin (figure 1*c*) is enriched on the oldest mitotic centrosome of the two centrosomes that make up the mitotic spindle [15,24,25], suggesting that while the full appendage structure may not be present, proteins that make up appendages are still organized based on centriole age and localized to the distal end of the mother centriole. In addition, cenexin is a known binding partner of Polo Like Kinase (PLK) 1 [12,13] and PLK1 is a known regulator of appendage formation [23]. One potential possibility for keeping cenexin at the right location on the centriole is that cenexin acts as a landmark for PLK1 to assist in the building of mother centriole appendages upon mitotic exit. The subdistal appendage protein centriolin [18,26] (figure 1*c*) redistributes between mitotic centrosomes during metaphase and forms a complex with the PCM component pericentrin [14]. The distal appendage protein CEP164 is also removed from the centrosomes during metaphase and to our knowledge no noted mitotic defects have been identified when CEP164 is depleted [15]. Taken together these studies suggest that subdistal and distal appendages are regulated by the cell cycle, but their molecular building blocks may have unique roles during specific cell cycle stages. Little to nothing is known about how CEP164 and centriolin are re-assembled at the mother centriole upon mitotic exit. Thus, it is important to understand the spatial and temporal localization of subdistal appendage proteins, with relation to the timeline of mother centriole maturation, to start creating a blueprint in appendage structure dynamics during cell cycle progression.

While it is argued that mother centrioles potentially do not have appendages during mitosis [23], there are several pieces of evidence that suggest appendage proteins play an important role in spindle placement. The mitotic spindle is a dynamic structure consisting of microtubules nucleated from the two mitotic centrosomes. The three classes of microtubules that make up the spindle are: (i) kinetochore microtubules, which attach to chromosomes involved in the separation of duplicated genetic material during anaphase; (ii) interpolar microtubules; and (iii) astral microtubules, which position and anchor the mitotic spindle to the cell cortex. Cortical landmarks, such as nuclear mitotic apparatus (NuMA) and the dynein–dynactin complex, connect astral microtubules with the cell cortex orchestrating how the spindle can orient within a cellular space and in relation to a developing tissue. Based on this, spindle orientation has been proposed to control cell fate choices, tissue architecture and tissue morphogenesis [27,28], and mother centriole subdistal appendage proteins have been implicated in these processes. For instance, ninein-null mice present with defects in spindle orientation during progenitor cell division that can result in altered progenitor cell numbers in mammalian skin [29]. Ninein is also important in the developing neocortex, where it has been shown to play a role in asymmetric centrosome inheritance and maintenance of progenitor cells through regulated spindle orientation [30,31]. Similarly, the appendage proteins centriolin [14] and cenexin [15] have been linked to regulating spindle positioning. Specifically, cenexin was linked to affecting a specific pool of microtubules, astral microtubules, that then influences spindle orientation and modulates NuMA localization at the cell cortex [15]. This regulation was shown to be required for apical–basal axis orientation and epithelial lumen positioning [15]. These studies highlight that there is an intrinsic asymmetry of the spindle that can be used to define cortical cues to direct spindle orientation and cell fate specification.

**Figure 3.**
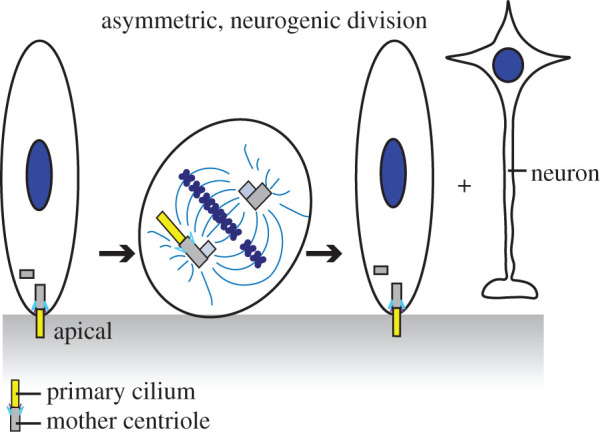
Asymmetric neuro divisions associate with a cilia remnant. The figure summarizes the relationship between a cilium in a neuro stem cell that will undergo an asymmetric neurogenic division (neurogenic divisions modelled from [33]). The dividing cell inherits a remnant of the cilium at the mitotic centrosome with the oldest mother centriole, which is usually the cell that remains stem and forms a cilium first. The other cell then goes on to differentiate.

Another potential regulator of cell fate specification is the primary cilium. A generally accepted model was that primary cilia are disassembled prior to mitosis so that centrioles can function at the poles of the mitotic spindle [32]. However, one study supported a model that inheritance of centrosome-associated structures (e.g. primary cilium) is involved in the asymmetric regulation of cell fate between daughter cells (figure 3). In the developing neocortex, a primary cilium extends from the apical membrane of epithelial neural stem cells into the lateral ventricle [34] (figure 3). Upon the onset of neurogenesis, apical progenitors switch from symmetric proliferative divisions to mainly asymmetric neurogenic divisions [33,35] (figure 3). When cells switch to asymmetric neurogenic divisions, the oldest mitotic centrosome (i.e. the one that is enriched with subdistal appendage proteins) is inherited into the daughter cell that is linked to maintaining stem cell character [31,36] (figure 3). Strikingly, the daughter cell that inherits the oldest mitotic centrosome does not completely disassemble its primary cilium prior to mitosis [37]. This study uncovered an unknown feature of cell division that gives insight to the relevance for asymmetric daughter cell behaviour that might explain the asynchrony in cilium re-establishment and potential asymmetries in signalling between daughter cells. However, this study also presents additional questions. For instance, does the mother centriole lose its appendages during mitosis in all cell types? Paridaen *et al*. [37] presented that prometaphase and metaphase neuronal stem cell's oldest centrosome (i.e. containing the oldest mother centriole) still contains mother centriole subdistal appendages along with a cilium remnant. This finding brings up an additional question, whether depletion of mother centriole appendage proteins that block the ability to form a cilium earlier on in the cell cycle results in later cell cycle defects that require the reabsorption of a primary cilium? Acute inhibition of subdistal appendage structure and function experiments are probably needed to clarify the role of subdistal appendages at different stages in the cell cycle.

## 3. The relationship between subdistal appendages and the midbody

A relationship between subdistal appendages and the final stage of cell division, severing of the cytokinetic bridge, has been identified. Cytokinesis and abscission are the final two stages of cell division, when a single cell is physically separated into two daughter cells. This separation is accomplished through the formation and ingression of a cleavage furrow. After furrow ingression, dividing animal cells stay interconnected for some time by a narrow intercellular bridge that contains a proteinaceous structure known as the midbody (figure 2*d*,*e*). The cleavage of this bridge is called abscission [38]. Before bridge cleavage, the mother centriole subdistal appendage protein, centriolin, localizes to the cytokinetic bridge, where it is thought to recruit the vesicle tethering complex, the exocyst [39]. Interestingly, this same complex was identified to localize to subdistal appendages in a centriolin-dependent manner [18]. With loss of centriolin or exocyst function, abscission failure ensues [26,39]. What is not known is whether centriolin can localize to both appendages and to the cytokinetic midbody during pre-abscission. Similarly, is this the case for the exocyst? Thus, with centriolin-loss is it centriolin's function at subdistal appendages that mediates abscission or its function at the cytokinetic midbody?

A potential mechanism to connect the centrosome with abscission may involve Rab11-mediated endosome trafficking. As mentioned previously, Rab11-endosomes, or recycling endosomes, can associate with mother centriole appendages mediated by centriolin and the exocyst [18]. Rab11-endosomes also transport with their associated cargo into the cytokinetic bridge, where these vesicles are thought to fuse and prime the membranes next to the midbody for an abscission event [40]. When inhibiting the ability of Rab11-associated vesicles to transport into the bridge using optogenetics, abscission failure occurs both in cell culture and in the zebrafish left-right organizer, Kupffer's vesicle [41]. In addition, during prometaphase and metaphase, Rab11-endosomes are thought to help build and maintain the growing PCM of the centrosome [42]. Interestingly, the Eps15 homology domain (EHD) 1 protein, known to regulate endocytic cargos exit from a Rab11 decorated recycling endosome compartment, has been implicated in removing PCM from the centrosome upon mitotic exit [43,44]. EHD1 and its interacting partner MICAL-L1 both localize to the centrosome and have been identified to promote ciliogenesis by removal of the mother centriole protein CEP110 [45,46]. However, how recycling endosomes and their associated regulatory proteins are coordinated to potentially interact with different sub-centrosome domains, such as the PCM and subdistal appendages, throughout the cell cycle remains unknown. An overarching theme does emerge where subdistal appendages and its associated molecular players, such as recycling endosomes, probably play a crucial role in cellular function, particularly coordinating abscission and subsequent cilia formation.

Another interesting aspect is whether a remnant of abscission, the cytokinetic midbody, could directly regulate centrosome function. Cytokinetic abscission requires membrane scission on either side, or sometimes both sides, of the cytokinetic midbody to occur. In certain cell types, the midbody can remain associated with one of the daughter cells and with time that midbody can move across the apical surface [47] (figure 2*d*,*e*). Transmission electron microscopy studies identified that the midbody remnant remains connected to the apical membrane by a membrane and microtubule-based tether. A model emerged from Bernabé-Rubio *et al.* [47] that the midbody could associate with the mother centriole to induce ciliogenesis. Creation and resolution of the cytokinetic bridge also involves many of the same components that are required for ciliogenesis, such as Rab11 [20,21], centriolin [26] and the exocyst [48]. Thus, could these proteins be released from the midbody and transported to the centrosome? The membrane and microtubule-based tether could provide a mechanism of transfer of these proteins to regulate centrosome maturation following abscission so that the centrosome is competent for cilia generation.

Studies suggest that the cytokinetic midbody is not always released after cytokinesis as reported in some cancer cells and differentiating stem cells [49,50], but instead the remains of the midbody may potentially have a function that is handled differentially by different cell types. In the *Caenorhabditis elegans* embryo a released cytokinetic midbody can act as a polarity cue to define the anterior–posterior axis [51]. In cancer cells, it was shown that post-abscission released midbodies can promote cell proliferation and anchorage-independent growth and survival [50]. Upon asymmetric severing of the cytokinetic bridge, the cell containing the oldest centrosome often retains the midbody remnant and that midbody remnant can contribute to the identity of the daughter cell [47]. Midbodies can selectively accumulate in stem cells, and midbody remnant loss can associate with stem cell differentiation [52]. How the oldest centrosome regulates whether the midbody should be retained is unknown. However, this is the centrosome that can make a cilium first between the two daughter cells [16] and is probably the centrosome that interacts with the midbody remnant in Bernabé-Rubio *et al.*'s study [47]. This mysterious connection between the cytokinetic bridge (e.g. midbody) and the centrosome will hopefully continue to unfold as we learn more about how each molecular and structural component functions.

## 4. Subdistal appendages: what do they do?

The subdistal appendages, once thought of as arbitrary structures, are now known for their ability to mediate anchoring, nucleation and release of microtubules. Foundational electron microscopy studies first suggested that microtubules terminate at subdistal appendages [53] and during interphase an aster of microtubules is formed around the mother centriole [54]. Additional studies showed that the interphase mother centriole is non-motile compared to the daughter [54], probably due to the aster of microtubules specifically around the mother centriole. Even though both centrioles are associated with γ-tubulin, the mother centriole is the only one that contains an aster of stabilized microtubules [54]. Piel *et al.* proposed that microtubules are nucleated near centriole walls in a γ-tubulin dependent manner, then released and transported to ninein-containing complexes associated with subdistal appendages where they are then anchored and become stabilized [54]. Aspects of this model were then further substantiated by removal of mother centriole appendages, through cenexin depletion resulting in loss of stabilized microtubule populations [15,55,56]. Stabilized microtubules are post-translationally modified and provide a potential ‘super-highway’ for motors to move on [57,58]. A variety of post-translational modifications are associated with stabilized microtubules, with most tubulin post-translational modifications being found at the outer surface of the microtubule [59]. However, one abundant post-translational modification that is decreased with loss-of-appendages is acetylation of a lysine residue that occurs within the lumen of a microtubule [15,18,55,60]. Interestingly, observations in cells noted that motors preferentially run along acetylated microtubules [57,61], but this was not confirmed within *in vitro* reconstitution experiments suggesting that microtubules possibly require additional post-translational modifications that occur *in vivo* to influence motor-function [62,63]. It will be interesting to investigate whether loss of mother centriole appendages also influences C-terminal derived post-translational modifications and whether these modifications affect motor-driven transport to the mother centriole.

Motor-driven transport to the centrosome is an essential process during the initial assembly of a primary cilium. Previous work identified membrane vesicles organized at the subdistal appendages of the mother centriole [64] and some of these vesicles were later identified to be Rab11-recycling endosomes [18]. Interestingly, these vesicles by transmission electron microscopy were shown to decorate the microtubules that emanated from the mother centriole appendages and also to directly associate with the appendages themselves [18]. Stabilized microtubules anchored at the subdistal appendages create an ideal pathway for Rab11-endosomes to deliver Rabin8 to the distal end of the mother centriole where it can activate Rab8 initiating ciliogenesis. Supporting this idea, the subdistal appendage protein cenexin was identified as a Rab8 effector protein [65]. Overexpression of cenexin and specific cenexin domains that bind to Rab8 blocked cilia formation relative to controls [65]. However, if subdistal appendages do indeed provide this function, then loss of subdistal appendages should lead to cilia defects. Deletion of the ODF2 locus (odf2^−/−^), which eliminates both Odf2 and its splicing variant cenexin1 that has a C-terminal extension, results in complete loss of mother centriole appendages causing failure in generating primary cilia [56]. Follow-up work went on to identify that cenexin is what is critical to ciliogenesis through its C-terminal extension that can bind to Rab8 and the further recruitment of Chibby, a centriolar component that is important for proper ciliogenesis [66].

While most of this work was done using *in vitro* cell culture systems (e.g. retinal pigment epithelial cells, mouse embryonic fibroblasts), recent advances have been made using the model organism, *Danio rerio* (zebrafish), in identifying the role of subdistal appendages in ciliogenesis. Mönnich *et al*. [67] identified the centrosome protein Cep128 (figure 1*c*) as a subdistal appendage protein required for ciliary signalling using a multi-functional approach of cell culture with CEP128 genomically removed, depleted using siRNAs, or depleting CEP128 transcripts in the zebrafish embryo using morpholinos. Interestingly, CEP128 loss does not overtly disrupt cilia structure, suggesting that subdistal appendages may be intact. However, CEP128 loss did grossly impair transforming growth factor- β/bone morphogenetic protein (TGF-β/BMP) mediated signalling events and defective Rab11-vesicle delivery of the TGF-β/BMP receptor to the base of the cilium [67]. This could possibly suggest that loss of Cep128 from appendages may affect microtubule docking at this site, disrupting targeted membrane transport to the mother centriole. Indeed, this seems to be the case where a follow-up study identified that loss of Cep128 is required for stabilizing centrosome-associated microtubule networks [68]. This study confirmed that Cep128 is at subdistal appendages in RPE and U2OS cells, and its loss caused the displacement of subdistal appendage proteins centriolin, ninein and Cep170 compared to control cells (figure 1*c*). Nevertheless, Odf2/cenexin remained localized to the distal end of the mother centriole [68]. These studies suggest that cenexin recruits and interacts with Cep128, which then establishes a hierarchical architecture of subdistal appendages required for anchoring microtubules through ninein. This study did not specifically examine the role of Cep128 in cilia formation, nor did it use transmission electron microscopy to examine the subdistal appendage structure. However, it does contribute to a potential model where subdistal appendages are required to create a specific stabilized microtubule network to the mother centriole that is potentially required for cilia formation and/or maintenance.

## 5. Conclusion: is the relationship between cilia and cell cycle progression mediated by subdistal appendages?

**Figure 4.**
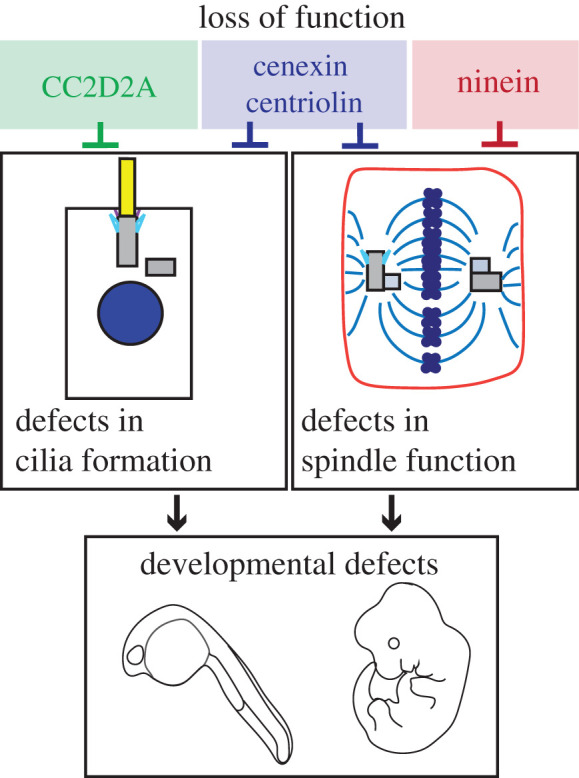
Appendage proteins result in cilia defects and/or spindle positioning defects with downstream consequences to development. CC2D2A (green) loss results in cilia loss, but no reported cell cycle defects. Cenexin and centriolin (blue) when lost cause defects in cilia formation and mitotic progression/spindle positioning, whereas loss of ninein (magenta) results in mitotic spindle defects (e.g. prometaphase/metaphase arrest and spindle positioning errors). Loss of any appendage protein (cenexin, CC2D2A, centriolin or ninein) results in severe developmental defects.

While subdistal appendages mediate both primary cilium formation and cell cycle progression, how these processes are potentially interconnected is understudied. It has become increasingly clear that understanding this potential connection may provide the needed insight to ciliopathy etiology. Ciliopathies are a class of disorders originally grouped together based on the ciliary localization and function of their causative proteins (some of which, like CC2D2A and pericentrin, are centrosome located, reviewed in [69]) and the observation that cilia are lost or dysfunctional in tissues from afflicted patients. However, different ciliopathy syndromes manifest in different organs, with varying severity, and at different stages of life. We argue that these differences may be partially explained by the multi-functional contributions of the centrosome to both cilia formation and cell cycle regulation. Thus, it is probably that ciliopathies could be caused by a complex set of disrupted functions resulting from defects in spindle orientation, cilia assembly/signalling, cell polarity and/or membrane trafficking, where defects in subdistal appendage structure/function could directly contribute to. This leads us to our last discussion, in which we aim to parse out the association between cilia formation/disassembly and cell cycle progression, and how this relationship may be mediated by the subdistal appendages. In order to understand this relationship, we are going to be assessing four centriole subdistal appendage proteins: cenexin, centriolin, ninein and CC2D2A (figure 1*c*; phenotypes modelled in figure 4).

Cenexin and centriolin, both known subdistal appendage proteins, have an identified role in both the cell cycle and in ciliogenesis [15,24,26,39,55]. Cenexin has been implicated in regulating spindle positioning and chromosome segregation [15]. During division, cenexin modulates preferential chromosome misalignment toward the oldest spindle pole in the event of mitotic error [24,55]. In zebrafish, a morpholino towards cenexin/ODF2 resulted in prolonged prometaphase consistent with problems in chromosome alignment, similar to what is observed in human cell lines [24,55,70]. One of these human cell lines, retinal pigment epithelial cells, also are unable to adequately form cilia when cenexin is lost [15]. In mice that express a truncated form of the mouse homologue, Odf2Δ/Δ, cilia are able to form, but functioning is perturbed resulting in mice presenting with ciliary dyskinesia [71]. These studies did not investigate potential defects in cell division. Centriolin, like cenexin, when depleted results in cilia loss and spindle orientation defects [14,26] (figure 4). Together, these findings suggest that some subdistal appendage proteins, such as centriolin and cenexin, may be multi-functional proteins that play many roles throughout the cell cycle, but it also could suggest that disrupting their role at one part of the cell cycle could lead to a domino of effects resulting in a handful of phenotypes. Specifically, that loss of cenexin/centriolin at one cell cycle point may cause phenotypes at a separate point during the cell cycle that is not explicit to a function for cenexin/centriolin at that specific cell cycle stage.

Understanding the role of centriolin in appendage function and downstream consequences also brings up an added layer of complexity. Where a potential splice variant of centriolin, CEP110 (also known as CCP110 or CP110), localizes to the distal end of centrioles and regulates ciliogenesis [72]. However, it has not been carefully examined when depleting CEP110 what happens to centriolin. With centriolin depletion it was reported that cilia were lost [26], and that with CEP110 depletion primary cilia become elongated [72]. If this depletion was indeed targeting the specific splice variant, CEP110, then it suggests that CEP110 may aid in the disassembly of the primary cilium before cell cycle re-entry can occur. However, in CP110^−/−^ mice, there was a reduction in primary cilia abundance in multiple tissues including the brain [73]. Mouse embryonic fibroblasts derived from CP110^−/−^ mice also displayed defects in mitotic progression consistent with a prometaphase/metaphase delay. These defects were associated with multi-polar spindles and monopolar spindle formation [73]. CP110^−/−^ mice die shortly after birth owing to organogenesis defects as seen in ciliopathies. The authors noted phenotypes of mutant pups were most similar to manifestations of short rib-polydactyly syndrome, a skeletal form ciliopathy in humans caused by mutations in diverse cilia-related genes [74]. Strikingly, in CP110^−/−^ mice, abnormal distribution of core components of subdistal appendages (e.g. ninein, CC2D2A) and in the localization of recycling endosomes (e.g. Rab11) occurred [73]. Rab11-endosome organization was previously reported to be regulated by the vesicle tethering complex, the exocyst, through its interaction with centriolin at subdistal appendages [18]. Molecular components that are associated with recycling endosomes, such as MICAL-L1 and EHD1, have also been linked to regulating the removal of CEP110 from the centriole to promote ciliogenesis [46]. Thus, could it be that both centriolin and CP110 were disrupted in these CP110^−/−^ animals or is it that CP110 has additional roles along with centriolin at subdistal appendages? While previous studies report the role of CP110 in suppressing the formation of cilia from the mother centriole [72], Yadav *et al*. [73] suggest a potential function of CP110 *in vivo* for promoting cilia formation. They present a testable hypothesis that CP110 has a context-dependent role during ciliogenesis that may be contingent upon available interacting proteins and the microenvironment within the cell, it will be interesting to further investigate this idea especially within *in vivo* contexts. Also, future studies are required to test the role of defective cell divisions identified in CP110^−/−^ animals contributing to disease pathologies.

Another subdistal appendage [75] and ciliary transition zone protein [76] that associates with recycling endosome associated MICAL-L3 and the small GTPase, Rab8, is CC2D2A. Mutations in CC2D2A (coiled-coil and C2-domains containing protein 2A) are the second most common genetic cause for the ciliopathy, Joubert syndrome [77,78], and can also result in the genetically related Meckel syndrome, which is a perinatal-lethal disorder characterized by encephalocele, polydactyly, cystic kidneys and liver fibrosis [79]. Using zebrafish, a chain of physical interactions linking CC2D2A to Rab8a through ninein-like protein (NINL) and MICAL-L3 occurs at the base of the cilium [77]. In murine models, CC2D2A^−/−^ mice resulted in embryonic lethality with multi-organ defects related to cilia biogenesis (figure 4). Importantly, CC2D2A loss prevents subdistal appendage assembly [75]. Here we propose a model, that like cenexin, CC2D2A may provide additional levels of regulation for cargo sorting at mother centriole appendages. Where Rab8-associated vesicles can dock through CC2D2A to potentially sort and direct cilium-targeted cargo proteins to the cilium itself. However, the role of CC2D2A in cell cycle progression has not yet been carefully examined.

The subdistal appendage protein, ninein, plays a critical role in the nucleation and anchoring of microtubules at the mother centriole [54], but during ciliogenesis it seems as though ninein may be dispensable. For instance, following ninein-loss, cilia are still able to assemble to a length similar to control cells [80]. Similarly, Lecland *et al.* [29] found no distinction between the presence of primary cilia within ninein-null and control murine tissues. Where ninein does seem to play an important role is in cell cycle progression and spindle positioning, with some similarities to what has been reported for both cenexin and centriolin (figure 4). For instance, ninein depletion resulted in a spindle positioning defect [29]. More severe cell cycle defects were also observed where cells can arrest in G2/M phase transition [81] or *Drosophila* embryos that express a loss-of-function homologue of ninein, Bsg25D, also tend to fail in prometaphase/metaphase resulting in large nuclear aggregates and failed embryogenesis [82]. Zebrafish embryos with ninein depletion also presented with microcephalic brain defects that were linked to cell cycle failure [83]. In summary, ninein's role during cell cycle entry is absolutely necessary for spindle function, whereas its role at appendages in interphase cells or during ciliogenesis is potentially supplementary to appendage function but is not required.

While major strides have been made in understanding the protein hierarchy and composition of these subdistal appendages, the understanding as to when the subdistal appendages begin construction and possibly disassemble with respect to the cell cycle is unknown. Having this understanding may provide needed insight in the multi-functional role of some of these subdistal appendage proteins during the cell cycle and during ciliogenesis.

## Data accessibility

This article has no additional data.
